# Abstracts and Items

**Published:** 1859-03

**Authors:** W. H. Byford

**Affiliations:** Chicago


### ABSTRACTS AND ITEMS.

PREPARED BY THE JUNIOR EDITOR.

Dr. E. A. Morrison, of Lawrenceville, Virginia, recommends, in the *Virginia Medical Journal*, quinine as the main item of treatment in scarlatina. He has used it for thirty-five years in the epidemic form, with the most beneficial results. He gives doses of a size suited to the different ages of the patients, every three hours from the beginning of the attack, with an occasional very mild cathartic, to keep the bowels soluble, and mops the throat with nitrate of silver in solution. He also, when the patient is old enough, recommends a gargle of red pepper tea.

IODIDE OF POTASSIUM, TO SUPPORT THE SECRETION OF MILK.

M. Roussel, the professor of clinical midwifery at Bordeaux, regards iodide of potassium as almost a specific in congested breasts during lactation. He gives it to the amount of six or eight grains a day. He thinks it suppresses the secretion and disperses the congestion with great certainty. He relates twenty cases of success, and none of failure. The above moderate amount, he thinks, does more good than larger quantities. The excessive secretion of milk may be prevented or moderated by giving it on the first or second day.—*Gazette des Hopitaux*, *North Carolina Journal.*

SANGUINARIA IN DYSMENORRHEA.

Dr. John D. O'Connor, of Sunfish, Ohio, in the *Lancet and Observer*, recommends the above article very highly in dysmenorrhea. His mode of administering it is to give a teaspoonful of the tincture three times a day, and a tablespoonful at bed time, commencing a fortnight before the period. If it does not succeed, he intermits its use until the same time in the next month. Observing the proper hygienic management, the remedy will succeed oftener than any other he has tried. Its full effects (which he desires to induce) are nausea, pain in the loins, extending through to the epigastric and iliac regions, as well as down the thighs.

PERSULPHATE OF IRON IN EPISTAXIS.

Dr. James F. Hibbard, of Richmond, Indiana, in the same journal, details the treatment of epistaxis by the above-named remedy. He throws an injection of a mixture formed of ten parts of water to one of the solution of the perrulphate, up the nostril from a glass syringe. He uses the solution recommended by Dr. Plummer, which is made according to the following formula, viz :

℞ Sulphate of Iron, - - ʒiiss.

Nitric Acid, - - - ℥iii.

Water, pure, - - - ℥xss.

Triturate the salt and acid together for fifteen minutes, then add the water and filter.

TRACHEOTOMY IN CROUP, VERSUS TUBING THE GLOTTIS.

It will be recollected that M. Bonchut has been urging tubing the glottis as a means of preventing asphyxia in croup. The Medical Society of the Hospitals of Paris has passed resolutions against this plan, and in favor of tracheotomy. The resolutions were offered by M. Behier. M. Trousseau says, operate (by tracheotomy) as soon as possible after formation of false membranes. M. Malgaigne thinks that we should try by all means to remove them, and upon failure to operate soon as possible. Bouillard thinks, 1st, that the surgical treatment of croup is fixed. Tracheotomy is the only proper operation. 2d. The medical treatment is to be remade. It should be, he thought, antiphlogistic. 3d. As to tubing the glottis, it is worthy of all the anathemas with which M. Trousseau has covered it.—*Gazette Hebdomadaire, from Lancet and Observer.*

GLYCEROLE OF ALUM IN ERYSIPELAS AND CHRONIC CUTANEOUS DISEASES.

The glycerole of alum is made in the following manner:

℞ Sul. alum reduced to an impalpable powder, ʒiss.
White precipitate, impalpable powder, grs. xv.

Mix; triturate well and pour into bottle, and add glycerine ʒxx. Shake the bottle until it assumes the consistence of cream.—*Presse Medicale Belge, Lancet and Observer.*

COUNTER-IRRITANTS, BY DR. THOMAS INMAN, OF LIVERPOOL, IN THE BRITISH MEDICAL JOURNAL.

1st. Counter-irritants, commonly in use, are direct irritants to the parts to which they are applied. 2d. Their acrid principle is absorbed, and acts in the milder form of a stimulant in the immediate neighborhood of its introduction. 3d. At last it enters the circulation, and affects distant organs. Acrid blisters, 1st, near the eye do harm in the acute stages of iritis, of lithalmia, and sclerotitis. They do good occasionally, in the latter stages, when the disease is chronic. 2d. Blisters to the throat are almost invariably prejudicial in the early stages of croup. 3d. Blisters do harm in the early stages of pleurisy, pneumonia, and pericarditis. They do good in the latter stages—at that period, in fact, in which we would use, could we apply direct means, the sulphate of zinc, nitrate of silver, etc., to the inflamed parts. 4th. They occasionally do good in bronchitis, but not under twenty-four hours after the blister has risen, and probably by the absorption of the stimulating material, and direct application through the circulation to the inflamed surface. 5th. Blisters are positively injurious in peritonitis, and in all its stages. They actually produce the disease in dogs and rabbits. 6th. They are equally injurious in recent gonorrhea and orchitis, very serviceable in chronic clap and swelled testicle. 7th. Blisters to the sacrum and copaiva internally have a very beneficial influence in leucorrhea. 8th. Blisters have a decided stimulating effect on the brain in the coma attending typhus or hydrocephalus.

In other words, blisters are prejudicial when the absorption of the stimulating principle injures the inflamed tissue by direct application. They do good in such cases as would be benefited by direct stimulation.

Again, 1st. There is no difference, except in degree, between the action of caustics and counter-irritants, generally, when applied to the unbroken skin. 2d. That these substances act intensely upon the part to which they are applied; more gently, but yet severely, upon the parts below and around them, and more mildly, yet still decidedly, upon the whole system. 3d. Blisters, etc., are useful only in those cases in which stimulants would be locally applied by the surgeon if the diseased parts were on the surface of the body, or within reach of his hand. 4th. Blisters are not essentially different in their modus operandi from such stimulants as iodide of potassium, copaiva, the warm balsams, essential oils, resins, etc., except in degree. 5th. That blisters are useful (in appropriate chronic cases) in proportion to the nearness of the diseased organ to the blistered surface. 5th. That, as a general rule, blisters have only a temporary influence; and that where they are really necessary and useful, they require to be repeated. 7th. The application of a vesicant irritant, or stimulating material, externally, involves the idea of there being local or systematic debility in the sufferer to correct, for which such stimulant is required. 8th. Counter-irritants of all kinds are physiologically incompatible with low diet, antimonials, purgatives, or other depressing remedies, inasmuch as it is manifestly absurd to stimulate locally and yet depress generally.

(To the above summary of Dr. Inman's ideas of counter-irritants, there is probably in the main not much objection, yet we think there are not many practitioners of experience who will unite with him in the proposition that they will agree in the character of their influence, and differ only in degree. We believe that each has its own peculiarities, and while they all stimulate locally and generally, each acts with more effect upon some particular tissue or organ, and that we should make choice between them as nearly as we can, in the present state of our knowledge. In many instances, of course, it may be indifferent. It is hardly probable, too, that oil of cantharides, after absorption, stimulates just like caustic potash.)—Ed.

### CURE OF SMALL POX IN 1306.

The learned physician, Gaddesden, at the court of Edward I., thus describes his practice in small pox: "I ordered the prince to be enveloped in scarlet cloth, and that his bed and all the furniture of his chamber should be of a bright red color; which practice not only cured him, but prevented his being marked;" and he states that he treated the sons of the noblest houses of England with the red system, and made good cures of all."—*Strickland's Lives of the Queens of England.*

